# Evaluation of environmental heat stress on physiological parameters

**DOI:** 10.1186/s40201-017-0286-y

**Published:** 2017-11-28

**Authors:** Zahra Zamanian, Zahra Sedaghat, Masoud Hemehrezaee, Farahnaz Khajehnasiri

**Affiliations:** 1 Department of Occupational Health, School of Health, Shiraz University of Medical Sciences, Shiraz, Iran; 2 Department of Epidemiology, School of Health, Shiraz University of Medical Sciences, Shiraz, Iran; 3 Department of Community Medicine, School of Medicine, Tehran University of Medical Sciences, Tehran, Iran

**Keywords:** Physiological parameters, Heat stress, Heat indices, Farmers

## Abstract

**Background:**

Thermal component of the atmospheric environment is an important issue which is related to human’s health. Thermal environment includes both heat exchange conditions (stress) and the physiological response (strain). The aim of this study was to measure the association of heat indices (PSI, HSI, Humidex) especially subjective one (STI) with some physiological parameters (Blood pressure, pulse rate and skin temperature).

**Methods:**

This is a cross-sectional study conducted on 387 male farmers on Boukan, West Azerbaijan, Iran in 2016. Sampling was conducted on the hottest days in summer on July based on the meteorological report. Heat parameters was measured 3 times in each session**.**

**Results:**

Direct associations were found between heat indices and physiological parameters except systolic BP. However, invers associations were found between blood pressure, skin and core body temperature, pulse rate with all heat indices. Based on the results of linear regression analysis, significant association was found between WBGT and skin temperature (B = 0.31, CI: 0.02, 0.61, *P* = 0.03). Results also showed significant association between Humidex and skin temperature (B = 0.21, CI: -0.03, 0.40, *P* = 0.02). However, no significant associations were found between other heat stress indices including UTCI, PHS, HIS, STI and Humidex with all study physiological parameters (core body temperature, systolic and diastolic blood pressure and also pulse rate).

**Conclusion:**

As expected, farmer’s health is affected by physiological parameters. Moreover, among assessed types of heat stress indices WBGT and Humidex were more powerful to show better the association with mentioned physiological parameters**.**

## Background

Thermal component of the atmospheric environment is an important issue which is related to human’s health [1]. Thermal environment includes both heat exchange conditions (stress) and the physiological response (strain) [1]. Heat is considered as an important environmental and occupational factor affecting humans^,^ health population with a wide range of physiological and psychological disorders including heat stroke, heat exhaustion, heat cramps, fainting, heat rash and heat fatigue, heart failure, kidney diseases, cardiac systolic disorder, and male’s infertility, depression and stress [2–6]. Due to workplace difficulties, suicide is highly common among those working in industry especially, farmers [7, 8]. For example, farmers in England and Wales appear to be one of the occupational groups with the highest risk of suicide, accounting for 1% of male suicides in the age group [9, 10].

Among workers with heavy jobs, farmers are the highest. In addition, agriculture is one of the most common industries with serious adverse effects of heat. As, farmers are exposed to outdoor heat extremes for a long time [11]. According to Mirabelli ‘s study among 161 heat related fatalities, 45% occurred in farmers [12]. In another study heat stress was responsible for approximately 40% of baseline symptoms including heat exhaustion, fainting and heat fatigue [13]. In Iran possibly due to hot and humid conditions, farmers are exposed to high rate of heat affecting their body functions including increase in blood pressure. Heart rate, oral and skin surface temperature [14–16].

In general, thermal stress indices can be divided into three groups according to their rationale: 1)indices based on calculations involving the heat balance equation (“rational indices”); for example, the Heat Stress Index (HSI) for warm weather required clothing insulations (IREQ) for cold environments, 2)indices based on objective and subjective strain (“empirical indices”); for example, the Physiological Strain Index (PSI), and WBGT 3)indices based on direct measurements of environmental variables (“direct indices”); for instance, dry resultant temperature (DRT) [17, 18].

Accordingly, International Standard Organization (ISO) 7243 (1989), suggested different reference values to keep the core body temperature bellow 38^°^c including Wet Bulb Globe Temperature (WBGT), the Heat Stress Index (HSI) indices based on objective and subjective strain (“empirical indices”); for example, the Physiological Strain Index (PSI) and also Humidex [19]. These values also depend on environmental factors, metabolic rate, and thermal insulation of cloth and work- rest regimens.

Potential hazards of working in hot environment depend on several physiological factors including increase in skin temperature, core body temperature and heart rate. The effect of man-environment heat exchange is subjectively felt as our response to the signals from heat and cool receptors distributed in the human skin. Skin temperature is an active component of the human heat balance. It depends on internal heat production and its transfer to skin surface, on ambient thermal stimuli and on the intensity of heat exchange.

Several studies are conducted to identify contributing factors to serious adverse effects of heat and the results came out conclusively in favor of a long list factors including age, clouting, history of disease, sex, level of physical activity and body size [20–23]. Despite numerous studies on the association of heat indices (especially WBGT) and physiological parameters, the association between other heat indices including PSI, HSI, Humidex and STI with physiological parameters is under debate [24–27]. This study is aimed to measure the association of heat indices especially subjective one (STI) with some physiological parameters (Blood pressure, pulse rate and skin temperature).

## Methods

This is a cross-sectional study conducted on 387 male farmers on Boukan (Latitude  36^°^31^'^13^"^N Longitude 46^°^12^'^31^"^E, Altitude m), West Azerbaijan, Iran in 2016. Sampling was conducted on the hottest days in summer on July based on the meteorological report. Heat parameters was measured three times in each session. Given hours and time intervals were determined via sharp change in temperature.

Climatic parameters were collected based on ISO-7726 and ISO-7243 as follows: T_nw_, and RH were measured by a humidity meter (model, REGD. DSGN. No. 917158). Additionally, air velocity (V_a_) was assessed using a digital flow meter (model, ST-8880) [28, 29]. In the next step, total metabolism was computed by summing up BMR and work metabolism [30]. Besides, the Mean Radiant Temperature (MRT) was calculated based on the following equation:$$ MRT=1.8\sqrt{V}\left({T}_a-{T}_g\right) $$. Dew point (dp) was also assessed according to the following equation:$$ dp={\left(\frac{f}{100}\right)}^{\raisebox{1ex}{$1$}\!\left/ \!\raisebox{-1ex}{$8$}\right.}\left(112+0.9{T}_a\right)+0.1{T}_a-112 $$). Also, metabolic rate was estimated based on ISO-8996 [30] [31]. Basal Metabolic Rate (BMR) was evaluated according to Harris-Benedict (1919).

Equation: (*BMR* = 66.4730 + (13.7516 × *Wb*) + (5.0033 × *Hb*) − (6.7550 × *A*) [32].

Environmental indices were assessed in this study as following:

(BMR = 66.4730 + 13.7516 × Wb + 5.0033 × Hb − 6.7550 × A).

### Wet Bulb Globe Temperature (WBGT)

To measure heat different heat indices were used. First, WBGT which was determined according to ISO7243 AND Burr (1991) (WBGT = 0.7tnw + 0.2tg + 0.1ta) [30]. Moreover, T_a_ was measured using a mercury thermometer, while black globe temperature (T_g_), WBGT time weighted average (TWA) was calculated following [33]:

$$ \mathrm{WBGT}\hbox{-} \mathrm{T}\mathrm{WA}=\frac{\left(\mathrm{WBGT}1\times \mathrm{T}1\right)+\left(\mathrm{WBGT}2\times \mathrm{T}2\right)+\cdots +\left(\mathrm{WBGT}\mathrm{n}\times \mathrm{T}\mathrm{n}\right)}{\mathrm{T}1+\mathrm{T}2+\cdots +\mathrm{T}\mathrm{n}} $$

WBGT-TWA: Time average of (C°) temperature indices.

T_n_: length exposure (hour).

### Universal Thermal Climate Index (UTCI)

**Fig. 1:**
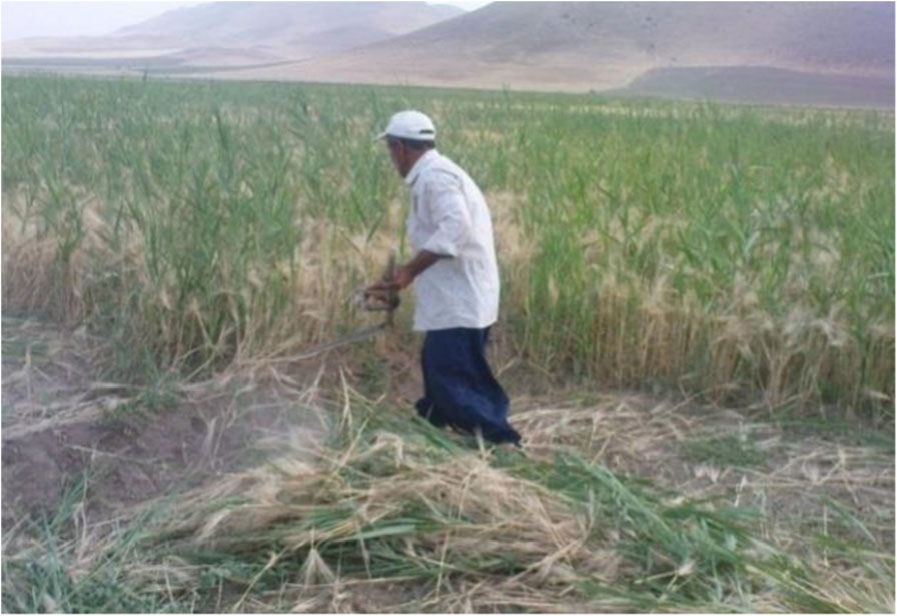
Farming in Buckan

The “Universal Thermal Climate Index” (UTCI) ultimately aims at developing a one-dimensional quantity, which adequately reflects the human physiological reaction to the multi-dimensionally defined actual thermal condition. The human reaction is simulated by a multi-node model of human thermoregulation [34], which is augmented by a clothing model. As illustrated in Fig. 1, the index value will be calculated from the multivariate dynamic output of that model [35].

The offset, i.e. the deviation of UTCI from air temperature depends on the actual values of air temperature (*ta*) and mean radiant temperature (*Tmrt*), wind speed (*v*) and water vapour pressure (*vp*). This may be written in general mathematical terms as:

UTCI = *f*(*ta*; *Tmrt*; *v*; *vp*) = *ta* + Offset(*ta*; *Tmrt*; *v*; *vp*).

### Subjective Temperature Index (STI)

It is an index that illustrates thermal load subjectively felt by man caused by ambient environment before the activation of adaptation processes. The STI depends both, on ambient conditions (temperature, solar radiation, wind, humidity) as well as on man-environment heat exchange [36].

### Heat Stress Index (HSI)

A heat stress index is another index measuring the effects of six basic parameters in any human thermal environment. The value is different from the thermal strain experienced by the person exposed to a hot environment. Such index value is used e to determine safe limits. Numerous studies are conducted to determine the definitive heat stress index. However, several debates are still ongoing) [37]. HSI is also calculated as following:

**Table:** 

Required components for calculating HSI index			
Index	Unit	Complete clothes	Incomplete clothes
Radiation	R (w/m^2^)	4.4 (35-t_r_)	7.3 (35-t_r_)
Conduction	C (w/m^2^)	4.6 V^0.6^(35-t_a_)	7.6 V^0.6^(35-t_a_)
Max Evaporation	E_max_ (w/m^2^)	7 V^0.6^(56-p_a_)	11.6 V^0.6^(56-t_a_)
		[Upper Limit of 390 (w/m^2^)]	
Required evaporation	E_req_ (w/m^2^)	E_req_ = M – R – C	
Heat stress Index	HSI	HIS = E_req_/E_max_ × 100	

### Predicted Heat Stress (PHS)

The aim of this study was to examine the performance of the current ISO PHS. This model is used to predict physiological response to the thermal environment.

In addition, PHS, is used to predict the time limit for those who are exposed to acute heat stress. [38]. As a result, persons can work safely under the heat stress conditions.

To evaluate protective validity, the time predicted via PHS is compared with SET.

### Humidex (HD)

The humidex (short for humidity index) is an index number used by Canadian meteorologists to describe how hot the weather feels to the average person, by combining the effect of heat and humidity. The humidex is a dimensionless quantity based on the dew point. It provides a number that describes how hot people feel, much in the same way the equivalent chill temperature, or “wind chill factor,” describes how cold people feel. Humidex is used as a measure of perceived heat that results from the combined effect of excessive humidity and high temperature [39].

Humidex value is an equivalent temperature measuring the (e actual temperature and the relative humidity of air. Its performance as following, in the dry weather it teaches to 30 °C but in the humid condition it falls down to 3 °C. This formula is very common in Canada [39].

humidex = (air temperature) + h

where, h = (0.5555)^∗^(e − 10.0); and,

e = 6.11^∗^ exp . (5417.7530^∗^((1/273.16) − (1/dew point)).

Also a related and simplified formula that takes temperature and the relative humidity inputs is:

Humidex = T + 5/9^∗^(e − 10)

*where* : *e* = *vaporpressure* (6.112 ∗ 10^∧^(7.5 ∗ *T*/(237.7 + *T*))^∗^ *H*/100).

T = air temperature (degrees Celsius).

H = humidity (%).

#### Data collection

Required data was collected using a questionnaire. The questionnaire consisted of some demographic characteristics. Physiological indices (blood pressure, pulse rate), type of activity (work posture and work clothing, color and type of clothing) and environmental data (dry-bulb temperature (T_a_), nature wet temperature (T_nw_), WBGT, HIS, STI, PSI, and Humidex) were measured. In addition, the physiological characteristics of the farmers were measured four times for each farmer in the warmest hour of the day. In addition, physiological parameters was measured in 4 steps including 7–9 A.M, 9–12 A.M, 12–15 P.M, and 15–16 P.M. Accordingly, oral temperature was measured via thermometer (the Beurer FT-09 model), heart rate, systolic and diastolic blood pressure was measured using an automated oscillometric sphygmomanometer (the Beurer BC-08 model). It is notifiable to mention that systolic BP is affected highly via environmental factors compared to diastolic BP. It is noticeable to mention that all the physiological parameters were measured and calculated based on ISO-9886 Standard.

#### Inclusion criteria

All patients were male, under 75 years of age**,** had no significant physical impairments and were in a stable condition.

### Study approval

An informed consent was read and signed by the participants before the interview was carried out.

### Statistical analysis

To conduct analysis, the study data were entered into the STATA software (version 16). Pearson’s correlation coefficient was used to determine the association between heat stress indices and other physiological parameters. Correlation between all study variables was measured.

Bivariate analysis was used to determine unadjusted associations between all above mentioned heat stress indices with some physiological parameters separately. Multivariate linear regression analysis was used to measure the adjusted associations of all independent variables with some physiological parameters. The modeling procedure was started after collinearity between the independent variables was measured using variance inflation factor index (VIF). The cut point for VIF was set at 10. Due to high collinearity of heat stress indices, separated models were used to measure the above associations.

## Results

In this cross-sectional sectional study, the participants were all male, predominantly less educated (52.87%), married (75.86%) and were in normal weight (60%), with on average 37.10 ± 14.22 years of age and 16.76 ± 12.16 years of experience and BMI 24.27 ± 4.17 kg/m^2^. Among participants 26.44% reported history of disease. Accordingly, only 4.60% were suffering from skin disease and kidney disease (Table 1). Significant differences were found in heat parameters including WBGT, UTCI, PHS, HSI, STI and Humidex between given times 7–9 PM, 9–12 AM, 12-15 PM, 15-16 PM and TWA (for all *p* < 0.001) (Table 2). As, significant difference in WBGT and HSI was found between all groups except (TWA and 12-15 PM, 15-16 PM and TWA) and (12-15 PM and 15-16 PM, 15-16 PM and TWA, 12-15 PM and TWA) respectively. Moreover, significant difference in UTCI, PHS and STI was observed between all groups except (TWA and 9–12 AM, 9–12 AM and 15-16 PM), (9–12 AM and 15-16 PM, 15-16 PM and TWA) and (9–12 AM, 12-15 PM) respectively. Accordingly, the highest value of stress heat was observed in UTCI, PHS and STI at 12–15 A.M. whereas, in WBGT, HSI and Humidex at 9–12 PM. Significant difference in WBGT were found. According to the Table 3 direct associations were found between heat indices and physiological parameters except systolic BP. However, invers associations were found between systolic blood pressure, skin and core body temperature, pulse rate with all heat indices.

**Table 1:** Demographic and clinical characteristic of participants

Variable		*N* (%)
Marital statues	Single	21 (24.14)
	Married	66 (75.86)
Education	Illiterate / primary	33 (37.93)
	Secondary/ tertiary	46 (52.87)
	High educated	8 (9.20)
BMI	< 25	51 (60)
	> 25	34 (40)
Second job	No	69 (79.31)
	Yes	18 (20.69)
History of disease	No	64 (73.56)
	Yes	23 (26.44)
History of medication	No	78 (89.66)
	Yes	9 (10.34)
Being in diet	No	82 (94.25)
	Yes	5 (5.75)
X ± SD		
Age (years)		37.10 ± 14.22
Work experience (years)		16.74 ± 12.16

**Table 2:** Comparison of different types of heat parameters in different times (X ± SD)

Time	WBGT	UTCI	PHS	HSI	STI	Humidex
7–9 PM	25.13 ± 1.22	24.62 ± 1.96	61.75 ± 16.49	14 ± 4.39	30.85 ± 5.78	29.43 ± 1.86
9–12 AM	29.03 ± 2.71	33.23 ± 5.09	135.52 ± 37.31	43.67 ± 37.31	43.67 ± 26.87	35.76 ± 4.21
12-15 PM	28.07 ± 0.69	35.86 ± 1.58	157.20 ± 11.63	37.1 + 16 ± 9.61	53.94 ± 4.66	34.99 ± 1.13
15-16 PM	27.32 ± 0.62	33.59 ± 0.84	129.69 ± 9.25	33.11 ± 6.87	43.25 ± 4.36	33.88 ± 0.83
TWA	27.65 ± 0.93	32.23 ± 1.17	125.71 ± 8.05	333.73 ± 6.20	47.50 ± 2.0	33.89 ± 1.38
*P*-value	<0.001	<0.001	<0.001	<0.001	<0.001	<0.001

**Table 3:** Correlation between all study variables (r)^a^

Variable	Skin temperature	Body temperature	Pulse rate	Systolic BP	Humidex	STI	HSI	PHS	UTCI	WBGT
Skin temperature	1.00	0.003	0.18	−0.18	0.25	0.02	0.11	0.11	0.12	0.24
Body temperature	0.003	1.00	0.18	−0.01	0.03	0.10	0.07	0.12	0.08	0.02
Pulse rate	0.18	0.18	1.00	−0.07	0.02	0.04	0.04	0.07	0.06	0.02
Systolic BP	−0.18	−0.13	−0.07	1.00	−0.004	−0.003	−0.004	−0.003	−0.002	−0.12
Humidex	0.25	0.25	0.02	−0.004	1.00	0.27	0.15	0.13	0.11	0.99
STI	0.02	0.10	0.04	−0.003	0.27	1.00	0.67	0.88	0.84	0.37
HSI	0.11	0.07	0.04	−0.004	0.15	0.88	1.00	0.72	0.90	0.11
PHS	0.11	0.12	0.07	−0.003	0.13	0.88	0.72	1.00	0.91	0.02
UTCI	0.12	0.08	0.06	−0.002	0.11	0.84	0.90	0.91	1.00	0.02
WBGT	0.24	0.02	0.02	−0.12	0.99	0.37	0.11	0.02	0.02	1.00

^a^Pearson correlation confident

According to the Table 4 unadjusted associations between WBGT (B = 0.34, CI: 0.05, 0.62, *P* = 0.02) Humidex (B = 0.23, CI: 0.04, 0.42, *P* = 0.01) and age (B = 0.02, CI: 0.001, 0.05, *P* = 0.03) with skin temperature were found. In addition, BMI (B = 0.02, CI: 0.002, 1.04, *P* = 0.04) and age (B = 0.15, CI: 0.003, 0.31, P = 0.04) had significant associations with systolic BP. The association of BMI and diastolic BP was significantly different (B = 0.02, CI: 0.002, 1.04, P = 0.04).

**Table 4:** Unadjusted association between some study variables with skin temperature, body temperature, systolic BP and diastolic BP

Skin temperature					Body temperature			Systolic BP			Diastolic BP		
Variable		β	CI	P.value	β	CI	P.value	β	CI	P.value	β	CI	P.value
Marital status	Single	–	–	–	–	–	–	–	–	–	–	–	–
	Married	0.34	−0.48,1.18	0.41	−0.03	−0.35,0.28	0.84	3.78	−1.34,8.91	0.14	4.52	−2.48,11.52	0.20
BMI		−0.01	−.10, −0.06	0.66	0.0005	−0.03,0.04	0.75	0.52	0.002,1.04	0.04	0.86	0.15,1.58	0.01
WBGT		0.34	0.05,0.62	0.02	0.01	−0.09,0.12	0.79	0.11	−1.71,1.94	0.90	−1.48	−3.95,0.99	0.23
UTCI		0.09	−0.08,0.28	0.28	0.02	−0.04,0.09	0.43	−0.01	−1.15,1.12	0.98	0.06	−1.48,1.61	0.93
PHS		0.01	−0.01,0.03	0.32	0.004	−0.003,0.01	0.28	0.003	−0.13,0.13	0.96	−0.0006	−0.18,0.18	0.99
HSI		0.04	−0.03,0.12	0.29	0.01	−0.02,0.04	0.47	0.04	−0.46,0.54	0.87	0.02	−0.66,0.72	0.93
STI		0.03	−0.05,0.06	0.92	0.01	−0.01,0.03	0.37	−0.01	−0.39,0.37	0.95	0.02	−0.50,0.54	0.93
Humidex		0.23	0.04,0.42	0.01	0.01	−0.06,0.08	0.72	0.04	−1.15,1.23	0.94	−1.002	−2.61,0.61	0.22
Age		0.02	0.001,0.05	0.03	−0.003	−0.01,0.005	0.41	0.15	0.003,0.31	0.04	0.03	−0.17,0.25	0.72
Experience work		0.02	−0.0005	0.05	−0.002	−0.01,0.008	0.67	0.11	−0.06,0.19	0.19	−0.008	−0.25,0.24	0.94

Based on the results of linear regression analysis, significant association was found between WBGT and skin temperature (B = 0.31, CI: 0.02, 0.61, P = 0.03) (Table 5). The results of Table 6 also showed significant association between Humidex and skin temperature (B = 0.21, CI: -0.03, 0.40, *P* = 0.02). However, no significant associations were found between other heat stress indices including UTCI, PHS, HIS, STI and Humidex with all study physiological parameters (core body temperature, systolic and diastolic blood pressure and also pulse rate).

**Table 5:** The adjusted association of heat pa rameter(WBGT) with skin temperature

Variable		β	CI	P.value
WBGT		0.31	0.02,0.61	0.03
Age		0.02	−0.02,0.7	0.33
Experience		0.006	−0.04,0.06	0.80
BMI		−0.03	−0.12,0.05	0.47
Marital status	Single	–	–	–
	Married	−0.09	−1.12,0.93	0.85

**Table 6:** The adjusted association of heat parameter(Humidex) with skin temperature

Variable		β	CI	P value
Humidex		0.21	−0.03,0.40	0.02
Age		0.02	−0.04,0.06	0.34
Work experience		0.009	−0.04,0.06	0.76
BMI		−0.02	−0.11,0.05	0.50
Marital status	Single	–	–	–
	Married	−0.12	−1.14,0.90	0.81

## Discussion

This cross-sectional study examined the association of heat stress and some physiological parameters. Based on the results, participants fundamentally young and only 16% of participants were older than 50 years of age. In comparison with other jobs the number of old individual working in agriculture is increasing so farmers would be more vulnerable to the adverse effects of heat [40]. Training farmers and their family seems to be urgent in this regard. As it mentioned above about 40% of participants were obese. It is possibly due to predominant change in agriculture process, less physical activity increases government’s concern on farmers’ heath which is in accordance with Dorner and Val davase’s studies [41, 42]. Obesity is considered as important contributing factor to be exposing to adverse effects of heat [42]. Accordingly, the more obesity is, the more heat disorders come with. As the result, all above mentioned factors were based on self- reported of participants at the time of interview which might affect farmers’ health.

Significant differences were found in heat parameters including WBGT, UTCI, PHS, HIS, STI and Humidex between given times 7–9 PM, 9–12 AM, 12-15 PM, 15-16 PM. Interestingly, all above heat parameters showed the highest value at noon which is related to increase and decrease in dry temperature and humidity respectively. Regarding PHS (physiological strain) and STI are subjective temperatures, job of the study population is categorized in moderate hot strain and hot condition respectively [36]. Farming is categorized as heavy workload and its WBGT-TWA value was (27.65 ± 0.93) in the present study [33]. Based on Iranian Occupational Exposure Limit (IOEL) category, half time rest and half time work is need. SIT is a subjective index and regarding the value of SIT is categorized as hot condition in the current study (47.5 ± 2). It is therefore crucial to manage the time and condition of working [36]. In this study PHS-TWA with value of (1.25 ± 0.8) is categorized as thermal slight strain. Humidex is also another important index used in this study measuring not only temperature but also humidity. Based on the Humidex Reading OSHA (Occupational Safety and Health Administration) the value of both Humidex-TWA (33.86 ± 1.38) and HSI-TWA (33.37 ± 0.2) are categorized as “some discomfort” so basic actions are needed to be done including drinking extra water and training workers about warning symptoms [39]. Regarding UTCI standard rang, this parameter is categorized as strong heat stress (32.23 ± 1.17) [35].

The results of the correlation between physiological parameters and heat indices showed invers associations between systolic blood pressure and all heat indices. As heat increases in the body, blood pressure reduces which is in accordance with Halonen’s study [43]. On the other hand, direct association was found between pulse rate and all heat parameters. Accordingly, increase in core body temperature positively affects pulse rate which is similar to Ren’s study [44]. Core body temperature is highly affected by heat stresses which it causes increase in dry and radiation temperature and finally makes sweeting which in turns to reduce skin temperature. As the result, the current study suggested that skin temperature would be a suitable parameter to assess heat stress.

In the present study, based on the final regression model WBGT and Humidex were significantly associated with skin temperature. Due to the fact that WBGT is affected by radiation and dry temperature, skin temperature is possibly affected by two above factors which is accordance with the Ramanathan’s results [45]. Humidex is also directly associated with skin temperature affected by relative humidity which in turns to change skin temperature [39].

## Conclusion

All measured physiological parameters including pulse rate, BP and core body temperature are health indices. As expected, farmer’s health is affected by physiological parameters. As a result, farmers’ health could be affected via heat stress and reduce their ability to perform different tasks. Moreover, among assessed types of heat stress indices WBGT and Humidex were more powerful to show better association with mentioned physiological parameters.
